# Preoperative predictors for return to physical activity following anterior cruciate ligament reconstruction (ACLR): a systematic review

**DOI:** 10.1186/s12891-023-06489-5

**Published:** 2023-06-09

**Authors:** Hayley M. Carter, Gwyn N. Lewis, Benjamin E. Smith

**Affiliations:** 1 Department of Physiotherapy, University Hospitals of Derby and Burton NHS Foundation Trust, Florence Nightingale Community Hospital, Derby, UK; 2 Centre for Rehabilitation and Ageing Research, Injury, Recovery and Inflammation Sciences, School of Medicine, University of Nottingham, Nottingham, UK; 3 Auckland University of Technology, Health and Rehabilitation Research Institute, Auckland, New Zealand

**Keywords:** Anterior cruciate ligament, Preoperative predictors, Return to physical activity, Return to sport

## Abstract

**Background:**

Rates of return to physical activity after anterior cruciate ligament reconstruction surgery are sub-optimal. Optimising presurgical treatment may improve return rates. The purpose of this systematic review was to identify modifiable preoperative predictors for return to physical activity after anterior cruciate ligament reconstruction.

**Methods:**

Seven electronic databases (CINAHL, MEDLINE and SPORTDiscus via EBSCOhost, AMED, PsycINFO and EMBASE via OVID and Web of Science) were searched from inception to 31 March 2023. The population of focus was adults aged 18–65 who had undergone primary anterior cruciate ligament reconstruction. Studies needed to identify at least one potential modifiable preoperative predictor variable and the relationship between the predictor(s) and return to physical activity. All time-points of assessment and study designs were included. Data extraction was completed by one reviewer and verified by a second reviewer. Two reviewers completed the risk of bias assessment using the Quality in Prognostic Studies tool and Grading of Recommendations Assessment, Development and Evaluation system.

**Results:**

The search identified 2281 studies, eight met the inclusion criteria. Five studies scored ‘high’, and three studies scored ‘moderate’ risk-of-bias. All preoperative predictors were of very low-quality evidence. Five different outcome measures were used to assess return to physical activity including Tegner, Marx, Physical Activity Scale, return to play at the elite level and return to preinjury level (undefined). This was measured between 1- and 10-years post-surgery. Nine preoperative physical, six psychosocial and five demographic/clinical factors were assessed and four were found to be predictive. These included quadriceps strength, psychological profile, patient estimated ability to return and graft type (patella tendon, BPTB).

**Conclusion:**

Very-low level evidence suggests that increasing quadriceps strength, managing patient expectations of their treatment outcomes, improving motivation to resume preinjury activity levels and considering the use of a BPTB graft will support return to physical activity after ACLR.

**Trial registration:**

This study was prospectively registered in PROSPERO: CRD 42020222567.

**Supplementary Information:**

The online version contains supplementary material available at 10.1186/s12891-023-06489-5.

## Background

Anterior cruciate ligament (ACL) injuries are a common musculoskeletal disorder among the adult population [1] with an estimated 200,000 ACL injuries occurring each year in the USA. [2] Most patients are treated surgically with an ACL reconstruction [3] (anterior cruciate ligament reconstruction, ACLR) following diagnosis. Those who do opt for surgery usually aim to return to their preinjury physical activity levels and expect this to be an achievable outcome. [4–7] However, this outcome is frequently documented to be sub-optimal, with only 24% returning at 1-year, [6] 28% at 18 months, [8] and less than 45% returning at 2-years. [9, 10] Psychological factors are the most commonly cited reason for failing to return to physical activity after ACLR. [11–13]

Understanding return to physical activity after ACLR remains a challenge. There is limited consensus on an agreed outcome measure to be used and time-frame for this to be assessed. [14] In 2016, a consensus statement suggested return to sport to be viewed as a continuum progressing from return to participation towards return to sport and finally return to performance. [15] A more recent consensus meeting in 2020 suggested a minimum two-year follow-up that records activity level and return to sport (among other outcomes). [14] However, a range of example tools were suggested for use and so no one universally agreed return to physical activity outcome exists. For the purpose of this review, we will use the umbrella term ‘return to physical activity (RTPA)’ to encapsulate all measures of return to physical activity/sport/preinjury level of activity.

Furthermore, there are a number of physical tests documented in the literature to support patients and clinicians to make decisions regarding the readiness and timing of RTPA, [16, 17] although the relationship between performance in these tests and returning to activity is not always clear. [16] Thus, clinical decision making remains a challenge.

ACLR is followed by an extensive postoperative rehabilitation period that typically lasts a minimum of 9 months and is usually progressed based on objective criteria. [18] What is less clear is how to manage patients in the preoperative period. There is typically a lengthy wait for an ACLR following injury in the UK National Health Service (NHS). This wait has been estimated at 4–12 months; [2] however, in recent years this has increased upwards of 12-months due to COVID-19 and the cancellation of elective procedures. [19, 20] Optimal treatment during this waiting time is currently unknown. [21].

Optimal presurgical treatment and clinical decision making regarding RTPA following ACLR could be facilitated by understanding preoperative variables that are associated with RTPA following surgery. This could aid hypothesis generation for novel treatment pathways to facilitate RTPA, allow clinicians to educate patients on the likely outcome of their surgery, and utilise appropriate outcome measures both pre- and post-operatively to track patients’ progress. As far as the authors are aware, no study to date has collated the evidence to identify modifiable preoperative predictors for RTPA after ACLR.

### Objective

The aim of this review is to identify the modifiable preoperative predictors for RTPA after ACLR.

## Methods

### Protocol and registration

This systematic review followed a published protocol [22] and was prospectively registered in PROSPERO (02 Dec 2020, https://www.crd.york.ac.uk/prospero/display_record.php?ID=CRD42020222567). The review is reported in line with the 2020 PRISMA checklist (available in supplementary file 1) [23] facilitated by the PERSiST guidance. [24]

### Eligibility criteria

The eligibility criteria were pre-specified by the Population-Exposure-Outcome-Study (PEOS) design and are described below.

#### Population

The population of focus was adults aged 18–65 years old who had undergone a primary ACLR with no concomitant injuries. Studies were included where participants were < 18 years but the mean age of the overall population was ≥ 18. Studies were included where participants had sustained a concomitant injury where results were separated.

#### Exposure

To be included, studies needed to identify at least one potential modifiable preoperative predictor variable and the relationship between the predictor(s) and RTPA. All estimates considered to determine the relationship between the predictive factor and outcome of interest were included (e.g., odds ratio and p-value). Predictive factors could be physical (e.g., quadriceps strength), psychosocial (e.g., anxiety) or demographic/clinical (e.g., graft type and time to surgery). We defined modifiable predictor variables as any factor that can be assessed and altered prior to surgery.

#### Outcome

The main outcome of interest was the success of RTPA. The identified preoperative risk factors needed to be linked to the outcome of interest. No time limit post-ACLR was defined for the reported outcome. If a study included multiple post-operative time-points, all were included. All measures of RTPA were included (e.g., participant reported [yes/no] or validated measures [Tegner, Marx scale]).

#### Study

Prospective, retrospective and cross-sectional study designs published in English with full texts available were included.

### Information sources

A pre-defined and published search strategy was followed and completed in December 2020 [22]. This search was carried out in six electronic databases in addition to screening the reference lists of included articles. This was updated in April 2023, in addition to expanding the search to an additional database (EMBASE).

### Search strategy

The search strategy included a combination of key words in four categories: (1) ACL, (2) preoperative, (3) risk factor and (4) RTPA. Terms were matched to Medical Subject Headings (MeSH) and combined using Boolean operators (e.g. Preoperative period OR Preop* OR Pre-op* OR Periop*).

### Selection process

Title and abstract screening was completed by one reviewer (HC). Two reviewers (HC and BS) independently screened full text articles for inclusion against the eligibility criteria. Agreement was discussed to reach consensus with discrepancies solved by the third reviewer (GL). Authors were contacted via the corresponding details if further information was required.

### Data collection process

Data extraction was completed by one reviewer (HC) in a form that was piloted prior to the review and described in the published protocol. [22] The accuracy of data extraction was verified by a second reviewer (BS).

### Data items

Extracted data included study design, participant details (number, age, sex), preoperative predictors, outcome measures and time point of outcome assessment post-surgery.

### Study risk of bias assessment

The Quality in Prognostic Studies (QUIPS) tool was used to assess for risk of bias for all included studies by two reviewers (HC and BS) independently. The QUIPS tool comprises of six domains to assess study participation, study attrition, prognostic factor measurement, outcome measurement, study confounding and statistical analysis and reporting. [25–27] A rating of low, moderate or high risk of bias is given for each domain to facilitate an overall risk of bias rating for each study. Disagreements between the reviewers were resolved through discussion.

### Certainty assessment

The Grading of Recommendations Assessment, Development and Evaluation (GRADE) system was also used to rate each predictor variable. GRADE has previously been adapted for prognostic research [28] to consider two factors that may increase the quality (moderate or large effect size and exposure-response gradient) and six factors that may decrease the quality (phase of investigation, study limitations, inconsistency, indirectness, imprecision and publication bias). This was therefore considered alongside the original framework. [29, 30]

The starting point of assessment for all studies was to determine the phase of investigation as advised by Huguet et al. [28] It was deemed all studies sought to identify associations between potential prognostic factors and the outcome of interest and so all started at a ‘moderate’ level of evidence. Publication bias was not assessed as funnel plot asymmetry is recommended to only be used when there are ten or more studies included. [31] Two reviewers independently rated each prognostic factor (HC and BS) and discrepancies were resolved through discussion.

### Synthesis methods

Clinical heterogeneity was assessed through visual examination of the data extraction table on details related to participant characteristics, risk factors, study design and processes in the included studies. Data were deemed to be heterogeneous due to the wide variety in study design, risk factors, reported outcome measures (e.g. Tegner and patient reported yes/no) and time to follow-up (e.g. 1 year, 2 years and 5–10 years) and so a narrative synthesis was completed.

Predictor variables were classified as physical, psychosocial, or demographic/clinical. For each variable, the number of studies it was investigated in and the number of studies showing a relationship to RTPA were determined. Predictor variables were classified as having a predictive, variable, or non-predictive relationship to RTPA based on a synthesis of these findings.

## Results

### Study selection

**Fig. 1:**
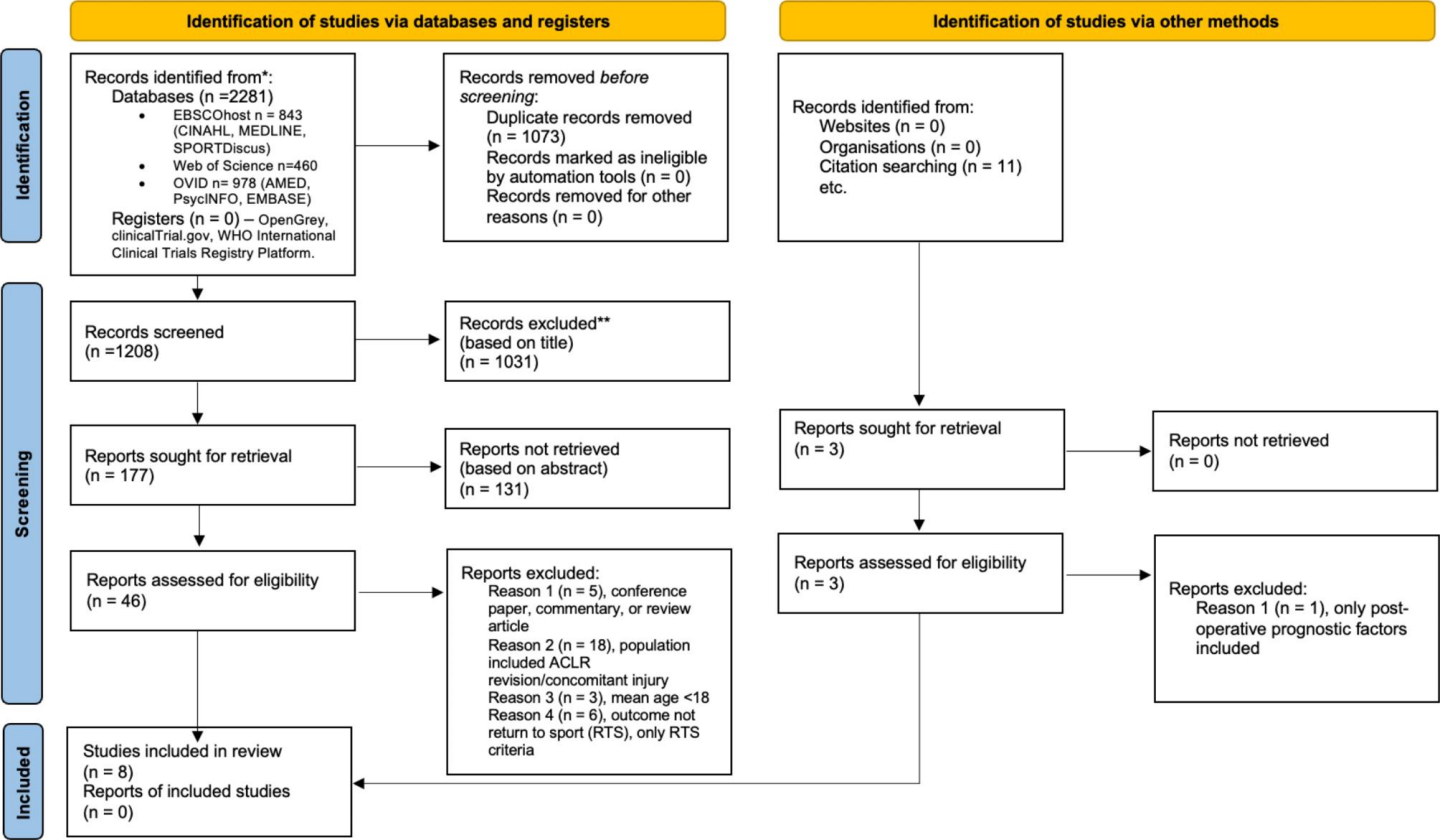
PRISMA 2020 flow diagram

The study selection process is presented in Fig. 1. The database search from inception to 31 March 2023 yielded 2281 articles. After duplicates were removed, 1207 articles were screened for inclusion. No additional articles were found from the screening of unpublished searches. After title and abstract screening, 46 full-text articles were assessed for eligibility and 40 were excluded due to study design (conference paper, commentary or review article), population (included concomitant injuries and mean age < 18), preoperative variables were deemed to be non-modifiable (e.g. age and sex), preoperative variables were not assessed against the outcome of interest and the outcome measure not related to RTPA. A further 11 potential articles were found from reference list screening. Two articles were deemed to meet the inclusion criteria (remaining articles excluded due to only including postoperative predictor variables, preoperative variables were deemed to be non-modifiable, population included concomitant injuries and preoperative variables were not assessed against the outcome of interest). The total number of studies included in the review was eight. [32–39]

### Characteristics of the included studies

The characteristics of the included studies are summarised in Table 1 and below.

**Table 1:** Study characteristics

Author	Year	Design	Participant details (n, mean age, % male)	Preoperative Predictors (Physical, Psychosocial and Demographic/clinical)	Outcome Measures	Time point of outcome assessment
Gobbi and Francisco	2006	Prospective	n = 100 Mean age: 28 % male: 67	**PHYSICAL** 1. **Knee scores** – Tegner, Noyes, and Marx 2. **Knee specific patient reported outcome measure’s** – Lysholm and International Knee Documentation Committee (IKDC) There were no significant differences in pre-operatively mean Tegner, Noyes, Lysholm and IKDC between those who return to sport (RTS) and those who didn’t RTS. The Marx scale showed a significant difference between athletes who were able to return to sport and those who did not return. It is however not clear from the reporting whether this was from the Marx scores taken pre-operatively or at final follow up. The authors were contacted but the corresponding email address provided on the article was no longer in use. This was therefore not included in the final analysis. **PSYCHOSOCIAL** 1. Psychovitality questionnaire* 28.6% of the athletes that ‘‘did not return to sport’’ scored ≤ 15 points preoperatively. Whereas, 67% of the athletes that ‘‘returned to sport’’, scored ≥ 15 points. Statistical analysis using the Mann–Whitney U-test revealed a significant difference (P < 0.001) between these two groups (Fig. 5a, b). **DEMOGRAPHIC/CLINICAL** n/a	Marx	Minimum of 24 months
Heijne, Äng and Werner	2009	Prospective	n = 64 Mean age: 30.1 % male: 45.3	**PHYSICAL** 1. **Passive knee range of motion (ROM)** 2. **Thigh muscle torque** (Concentric quadriceps* and hamstrings, Eccentric quadriceps and hamstrings) 3. **Knee function (2 questions from IKDC)** 4. **Anterior knee pain** **PSYCHOSOCIAL** n/a **DEMOGRAPHIC/CLINICAL** 1. **Weight and BMI** 2. Graft type* 3. Time to surgery* Three pre-operative variables were correlated with the Tegner activity scale: 1. Concentric quadriceps torque (P = 0.004) 2. Graft type – patella tendon (P = 0.006) 3. Time between injury and reconstruction surgery (P = 0.02) (Table 3). These four variables explained 21% (P = 0.006) of the outcome variance. The use of a patellar tendon graft in favour of a hamstring graft was shown to be the strongest predictor in the forward step-wise regression analysis, but explained only 8% of the outcome variance (Table 4).	Tegner	12 months (range 12–16)
Hetsroni et al.,	2017	Retrospective	n = 55 Mean age: 25.3 % male: 100	**PHYSICAL** 1. **Physical activity rating scales** - Tegner* and Marx Standard correlation coefficient for Tegner preinjury score as the independent variable and Tegner at follow-up as the dependent variable β^b^ 0.423 (0.437), p = 0.01 (< 0.01). Standard correlation coefficient for Tegner preinjury score as the independent variable and Marx at follow-up as the dependent variable β^b^ 0.405 (0.457), p = 0.02 (< 0.01). There was no correlation between the Marx pre-injury scores and follow-up Marx and Tegner scores. Standard correlation coefficient for the Marx pre-injury score as independent variable and Marx follow-up as the dependent variable β^b^ -0.088, p = 0.6 and for Tegner follow-up as the dependent variable β^b^ -0.164, p = 0.29. **PSYCHOSCOCIAL** n/a **DEMOGRAPHIC/CLINICAL** 1. **Time to surgery** 2. **Smoking status** There was no association between time to surgery and smoking status with the outcome measure.	Tegner Marx	5–10 years (mean 7.1 years)
Jurkonis, Gudas and Smailys	2018	Prospective	n = 214 Mean age: 33.2 % male: 74.3	**PHYSICAL** 1. **Physical activity rating scale** - Tegner* A positive correlation was found between pre-injury Tegner activity score and Tegner activity score 12 months after surgery (r = 0.728, p < 0.001) **PSYCHOSCOCIAL** n/a **DEMOGRAPHIC/CLINICAL** 1. BMI* A multiple regression analysis indicated that a model including age, sex, BMI and pre-injury Tegner was a significant predictor of Tegner 12-months after ACLR, BMI (*b*_2_= -0.042, *p* = 0.008). Table 3 shows Tegner activity score pre-injury and at 12 months after ACLR in BMI groups, with statistically significant differences between Tegner activity scores in BMI groups.	Tegner	12 months
Liptak and Angel	2017	Retrospective	n = 115 Mean age: 24 % male: 100	**PHYSICAL** n/a **PSYCHOSCOCIAL** n/a **DEMOGRAPHIC/CLINICAL** 1. Weight* Player weight was identified as significantly associated with returning to play at the elite level. Heavier players displayed a tendency to be more likely to return to play at the elite level when compared with lighter players (70–79 kg). Players weighing between 90 and 99 kg were nearly 15 times more likely to return to play at the elite level (OR, 14.93; 95% CI, 2.13-104.76; P = 0.007).	Return to play at the elite level (AFL Medical Officers Association [AFLMOA] data)	No pre-defined time of assessment but documented as < 1 year after injury or 1 year or longer
McGrath et al.,	2016	Prospective	n = 64 Mean age: 26.9 (LARS group) and 28.9 (2ST/2GR group) % male: 68.8	**PHYSICAL** 1. **Physical activity rating scale** - Tegner* Preoperative Tegner scores had a moderate correlation with postoperative activity levels at 12 (r_s_ = 0.347, P = 0.005) and 24 months (r_s_ = 0.326, P = 0.009). **PSYCHOSCOCIAL** n/a **DEMOGRAPHIC/CLINICAL** n/a	Tegner	12 and 24 months
Sonesson et al.,	2016	Prospective	n = 65 Median age: 22 % male: 52.3	**PHYSICAL** n/a **PSYCHOSCOCIAL** 1. **Estimation** – Time to achieve postoperative markers and ability to return to preinjury level* There were no differences between participants who had returned to their pre-injury sport activity at 1 year and those who had not returned, regarding preoperative estimated time to: - complete rehab (n.s) - be able to run/jump (n.s) - return to pre-injury sport activity (n.s) However, participants who had returned to their pre-injury sport activity at 1 year, to a greater extent estimated pre-operatively that it was possible to return to their preinjury activity level compared to those who had not returned at 52 weeks (P = 0.019, Table 4). 2. **Goals** Returning to preinjury sport activity at 1 year was not related to whether participants said their goal was to return preoperatively (n.s). **DEMOGRAPHIC/CLINICAL** n/a	Return to preinjury level (unspecified)	12 months
Thomeé et al.,	2008	Prospective	n = 38 Mean age: 29.7 % male: 65.8	**PHYSICAL** 1. **Physical activity rating scale** - Tegner* Tegner_Pre−injury_ was a significant predictor (P = 0.002, β = 0.28) of Tegner_Present_ at the 1-year follow-up when adjusted for age, gender, K-SES_Present_ and K-SES_Future_ (R^2^ = 0.25). **PSYCHOSCOCIAL** 1. K-SES present* The pre-operative K-SES_Present_ correlated significantly (P = 0.03) (r_s_ = 0.37) with Tegner_Present_ at the 1-year follow-up. But did not correlate significantly (P = 0.20) (r_s_ = 0.32) with PAS_Present_ at the 1-year follow-up. 2. **K-SES future** The pre-operative K-SES_Future_ did not correlated significantly (P = 0.56) (r_s_ = 0.10) with Tegner_Present_ or PAS_Present_ P = 0.76) (r_s_ = 0.01) at the 1-year follow-up **DEMOGRAPHIC/CLINICAL** n/a	Tegner Physical Activity Scale (PAS)	12 months

*Predictive factor

### Study designs

Of the eight studies, six were a prospective design and the remaining two were retrospective. The included studies were published between 2006 and 2018 in five different countries (Australia, Italy, Israel, Sweden and Lithuania).

### Participants

The studies investigated a total of 715 participants. Sample sizes ranged from 38 to 214. The age of participants ranged from 12 to 64 years. The overall mean age was > 18 for seven studies with one study reporting a median (22 years) only. Females were underrepresented among the total participants (n = 187, 26.15%), with two studies excluding female participation. [34, 36]

The population of two studies included concomitant injuries (meniscal injuries requiring surgery). [32, 34] However, both stated that inclusion of these participants did not impact the results.

#### Predictive factors

A total of 18 predictive factors were assessed across the eight studies (Table 2), which are discussed below.

**Table 2:** Predictive factors investigated across the eight studies

Predictor Variable		
Physical	Psychosocial	Demographic / Clinical
Physical activity rating scale (Tegner,^32,34,35,37,39^, Noyes^32^ and Marx^34^)	Psychovitality questionnaire^32^ (score ≥ 15)	Body Mass Index (BMI)^33,35^
Knee specific patient reported outcome measure (Lysholm,^32^ International Knee Documentation Committee [IKDC]^32^ and ‘knee function’^33^ (defined as 2 questions from IKDC)	Knee-Self Efficacy Scale (K-SES) Present^39^	Weight^33,36^
Passive knee range of motion^33^	K-SES Future^39^	Time to surgery^33,34^
Concentric quadriceps torque^33^	Estimation of ability to return to preinjury level^38^	Graft type (bone-patella tendon-bone)^33^
Eccentric quadriceps torque^33^	Estimation of time (number of months) to achieve postoperative markers^38^	Smoking status^34^
Concentric and eccentric hamstring torque^33^	Goal to return to preinjury level^38^	
Anterior knee pain^33^		

#### Physical factors

Physical factors were assessed in six of the eight studies. There were three scales used to assess physical activity engagement: Tegner activity scale (assessed in five studies^32,34,35,37,39^), [40] Noyes (assessed in one study^32^) [41] and Marx scale (assessed in one study^34^). [42] There were also three knee specific patient reported outcome measures: Lysholm score (assessed in one study^32^), [43] International Knee Documentation Committee (IKDC) (assessed in one study^32^), [44] and ‘knee function’ which asked two questions from the IKDC (assessed in one study^33^). The remaining measures were physical assessments of knee/lower limb function and pain (all assessed in one study^33^), including passive range of motion (ROM), muscle strength (torque) and anterior knee pain.

#### Psychosocial factors

Psychosocial factors were assessed in three of the eight studies. One study [32] produced a bespoke outcome measure called ‘psychovitality’ which was described as a psychological profile questionnaire. In this questionnaire, psychological factors including patients’ expectations relating to treatment outcomes and motivation to resume pre-injury activity levels were assessed. Scores could range from three to 18 points, with a higher score indicating higher levels of motivation of the patient.

The second study [39] used the Knee Self-Efficacy Scale (K-SES) which is a 22-item scale used to assess a patients perceived self-efficacy under four headings: (1) daily activities, (2) sports and leisure activities, (3) physical activities and (4) your knee function in the future.

The final study [38] asked patients to estimate the number of months it would take to achieve a number of postoperative markers (complete rehabilitation, be able to run/jump and return to their preinjury sport activity), their ability to return to their preinjury level of activity, and whether their goal was to return to their preinjury level.

#### Demographic/clinical factors

Demographic and clinical factors were assessed in four of the eight studies. This included body mass index (BMI; assessed in two studies [33, 35]), weight (assessed in two studies [33, 36]), time from injury to surgery (assessed in two studies [33, 34]), graft type [33] and smoking status. [34].

#### Outcome measures

Five different outcome measures were used across the eight studies (Table 3): (1) Tegner (2) Marx (3) Physical Activity Scale (PAS) (4) Return to play at the elite level (5) Return to preinjury level (the authors did not specify how this was measured and did not respond to our email asking for clarification). These were measured at a range of different time-points from 1 to 10 years post-operatively.

**Table 3:** Outcome measures used and time-point of assessment

Study	Outcome Measure	Time-point of Assessment
Gobbi and Francisco	Marx	Minimum of 24 months
Heijne, Äng and Werner	Tegner	12 months (range 12–16 months)
Hetsroni et al.,	Tegner Marx	7.1 years (range 5–10 years)
Jurkonis, Gudas and Smailys	Tegner	12 months
Liptak and Angel	Return to play at the elite level (AFL Medical Officers Association [AFLMOA] data)	No pre-defined time of assessment but documented as < 1 year after injury or 1 year or longer
McGrath et al.,	Tegner	12 and 24 months
Sonesson et al.,	Return to preinjury level (unspecified)	12 months
Thomeé et al.,	Tegner Physical activity scale (PAS)	12 months

### Risk of bias assessment

A summary of the risk of bias assessment, using the QUIPS tool, is shown in Table 4. Of the eight studies, five assessed as high risk of bias and three assessed as moderate risk of bias. Percentage agreement between the two reviewers (HC and BS) for the individual risk of bias domains for the QUIPS tool was 88%. Cohen’s kappa statistic, indicating the agreement between reviewers, was k = 0.82, which is considered almost perfect. [45] All discrepancies were resolved through discussion.

**Table 4:** QUIPS scores showing risk of bias

	Study participation	Study attrition	Prognostic factor measurement	Outcome measurement	Study confounding	Statistical analysis & reporting	Overall Rating
Gobbi and Francisco	Low	High	Moderate	Moderate	High	Moderate	**High**
Heijne, Äng and Werner	High	Low	Moderate	Low	High	Moderate	**High**
Hetsroni et al.,	Moderate	Moderate	Moderate	Low	High	Moderate	**Moderate**
Jurkonis, Gudas and Smailys	Moderate	High	Low	Low	High	Moderate	**High**
Liptak and Angel	Low	Low	Moderate	Moderate	High	Moderate	**Moderate**
McGrath et al.,	Moderate	High	Moderate	Moderate	High	Moderate	**High**
Sonesson et al.,	Low	High	Moderate	Moderate	Moderate	Moderate	**Moderate**
Thomeé et al.,	High	High	Moderate	Low	Moderate	Moderate	**High**

### Certainty of evidence

A summary of the GRADE assessment for each predictive factor is shown in Table 5.

**Table 5:** GRADE scores for each predictive variable

Summary of Results		GRADE Assessment						
		Factors that may decrease the quality				Factors that may increase the quality		Quality
Preoperative Predictive Factor	Number of Participants (studies)	Study Limitations	Inconsistency	Indirectness	Imprecision	Moderate/large effect size	Dose effect	
Physical activity rating scale 1) Tegner 2) Marx	403 (4)	High/moderate risk of bias using QUIPS	Inconsistency^b^	Indirectness^c^	No imprecision	No moderate or large effect size	No exposure-response gradient	$\oplus$◯◯◯ Very low
Knee specific patient reported outcome measure 1) Lysholm 2) IKDC 3) ‘Knee function’ (measured using two questions from the IKDC) 4) Noyes	164 (2)	High risk of bias using QUIPS	Inconsistency^b^	Indirectness^c^	Imprecision^e^	No moderate or large effect size	No exposure-response gradient	$\oplus$◯◯◯ Very low
Passive knee range of motion	64 (1)	High risk of bias using QUIPS	Inconsistency^a^	Indirectness^d^	Imprecision^e^	No moderate or large effect size	No exposure-response gradient	$\oplus$◯◯◯ Very low
Thigh muscle torque	64 (1)	High risk of bias using QUIPS	Inconsistency^a^	Indirectness^d^	Imprecision^e^	No moderate or large effect size	No exposure-response gradient	$\oplus$◯◯◯ Very low
Anterior knee pain	64 (1)	High risk of bias using QUIPS	Inconsistency^a^	Indirectness^d^	Imprecision^e^	No moderate or large effect size	No exposure-response gradient	$\oplus$◯◯◯ Very low
Weight	179 (2)	High/moderate risk of bias using QUIPS	Inconsistency^b^	Indirectness^c^	Imprecision^ef^	No moderate or large effect size	No exposure-response gradient	$\oplus$◯◯◯ Very low
BMI	278 (2)	High risk of bias using QUIPS	Inconsistency^b^	Indirectness^c^	Imprecision^g^	No moderate or large effect size	No exposure-response gradient	$\oplus$◯◯◯ Very low
Graft type	64 (1)	High risk of bias using QUIPS	Inconsistency^a^	Indirectness^d^	Imprecision^e^	No moderate or large effect size	No exposure-response gradient	$\oplus$◯◯◯ Very low
Psychovitality	100 (1)	High risk of bias using QUIPS	Inconsistency^a^	No indirectness	Imprecision^e^	No moderate or large effect size	No exposure-response gradient	$\oplus$◯◯◯ Very low
Estimation of time (number of months) to achieve postoperative outcomes markers	65 (1)	Moderate risk of bias using QUIPS	Inconsistency^a^	No indirectness	Imprecision^e^	No moderate or large effect size	No exposure-response gradient	$\oplus$◯◯◯ Very low
Goal to return to preinjury level	65 (1)	Moderate risk of bias using QUIPS	Inconsistency^a^	No indirectness	Imprecision^e^	No moderate or large effect size	No exposure-response gradient	$\oplus$◯◯◯ Very low
Estimation of ability to return to preinjury level	65 (1)	Moderate risk of bias using QUIPS	Inconsistency^a^	No indirectness	Imprecision^e^	No moderate or large effect size	No exposure-response gradient	$\oplus$◯◯◯ Very low
Knee Self-Efficacy Scale (K-SES)	38 (1)	High risk of bias using QUIPS	Inconsistency^a^	No indirectness	Imprecision^e^	No moderate or large effect size	No exposure-response gradient	$\oplus$◯◯◯ Very low
Time to surgery	119 (2)	High/moderate risk of bias using QUIPS	Inconsistency^b^	Indirectness^c^	Imprecision^g^	No moderate or large effect size	No exposure-response gradient	$\oplus$◯◯◯ Very low
Smoking status	55 (1)	Moderate risk of bias using QUIPS	Inconsistency^a^	Indirectness^d^	Imprecision^e^	No moderate or large effect size	No exposure-response gradient	$\oplus$◯◯◯ Very low

^a^Only single trial available

^b^Estimates of the predictive factors association with the outcome vary in direction and/or no confidence intervals available to assess overlap

^c^Variety in the outcome measures used and time-points at which they were assessed

^d^The final sample only represents a subset of the population of interest

^e^Small sample size < 400 – single study

^f^Variety in time-points at which the preoperative factor is assessed

^g^Small sample size < 400 – multiple studies

All preoperative variables were assessed to be very low-quality. All factors were downgraded for (1) study limitations, as all studies were graded as ‘high’ or ‘moderate’ risk of bias using the QUIPS tool, (2) inconsistency, as only a single trial was available for most predictive factors or, where more than one trial was available, there was inconsistency in association between the predictive factor and the outcome of interest and no confidence intervals were available to assess overlap. Some factors were downgraded for indirectness where it was determined that the study population was unlikely to be representative of the entire population of interest or where outcome measures and time-points of assessment differed between studies assessing the same preoperative factor. Only one variable was not downgraded for imprecision as the combined sample size of the studies it was assessed in was greater than 400 participants. No factor was deemed to fit the criteria to increase the quality in either of the two categories.

### Study outcomes

Of the 18 predictor variables, one physical factors (high concentric quadriceps torque), two psychosocial factors (high psychovitality, and positive estimation of ability to return to preinjury level) and one demographic/clinical factor (bone-patella tendon-bone graft, BPTB) were found to be positively associated with RTPA between one and ten years post-ACLR. Six physical factors (knee specific patient reported outcome measure, pivot shift, passive knee range of motion, eccentric quadriceps torque, concentric and eccentric hamstrings torque and anterior knee pain), three psychosocial factors (K-SES_Future_, patient estimation of time to achieve postoperative markers and goal to return to preinjury level) and one demographic/clinical factor (smoking status) were not found to be associated with RTPA within ten years of an ACLR. There were five factors (one physical: high physical activity recorded on a rating scale, one psychosocial: higher K-SES_Present_ and three clinical/demographic: low BMI, weight and time to surgery) with variable results as to whether they were predictive or not predictive of returning. The 18/20 variables are summarised in supplementary file 2, grouped by the categories discussed above.

## Discussion

### Summary of main findings

The available evidence identifying modifiable preoperative predictors of RTPA following ACLR is poor in both quantity (evidenced by only eight studies eligible for inclusion) and quality (high and moderate risk of bias and very low-quality according to GRADE). A total of 18 potential predictor variables were identified, of which four were found to be predictive of returning to physical activity after ACLR. Identified areas include: quadriceps strength (concentric quadriceps torque), psychovitality, patients’ estimation of their ability to return to their preinjury level and graft choice. However, the QUIPS tool and GRADE framework challenge the certainty of these results.

### Clinical and research implications

In the absence of formal guidelines to direct the preoperative stage of patient care the four identified factors in this review support clinical decision making by offering direction for this stage of treatment. Firstly, our findings suggest that, for patients aiming to RTPA, clinicians delivering preoperative treatment should aim to increase quadriceps strength, improve psychovitality (by addressing patients’ expectations related to treatment outcomes and motivation to resume pre-injury activity levels) and understand patients’ estimations of their postoperative outcomes. Secondly, evidence has been presented to support the BPTB graft in facilitating RTPA, adding to the discussion of optimum graft choice.

Whilst awaiting surgery, preoperative rehabilitation (also termed ‘prehabilitation’) has been identified as an important component to help patients prepare, both physically and mentally, for surgery and postoperative rehabilitation. [46–48] However, clinical practice for this stage of treatment varies. [49] Typically, prehabilitation research to date has concentrated on physical aspects alone, which is likely to provide limited benefit [21] given the variety of preoperative variables identified outside the ‘physical’ category. Quadriceps strength (concentric quadriceps torque) was highlighted as the only physical factor predictive of RTPA. Improving quadriceps strength is a commonly cited target following ACL injury and has previously been included in ACLR prehabilitation programmes. [21, 50, 51] Strength training principles would suggest a high-intensity, low repetition programme; however, in a pre-surgical ACL injured population this may need to be adapted to meet tolerable levels of pain, physical symptoms (e.g. joint effusion) and/or patients’ lifestyle factors dictating their availability to engage in rehabilitation. There is limited consensus to determine the optimal length of prehabilitation programmes, [21, 49] but strength training literature suggests a minimum 3-month programme which is appropriate for implementation in the NHS preoperative time-frame.

Two psychosocial factors, high psychovitality and patients’ positive estimation of their ability to return to preinjury level of sport, were identified in our review as having a positive relationship with RTPA. Psychovitality was measured using a bespoke questionnaire designed to assess patients’ expectations related to treatment outcome and motivation to resume pre-injury activity levels. Patients’ preoperative expectations have previously been linked to motivation to engage in rehabilitation and overall satisfaction. [38, 52, 53] Understanding patient expectations should form an integral part of a clinical assessment and setting realistic expectations of the outcome of ACLR surgery is an important component of patient care. [54] Patient expectations of ACLR treatment outcomes has previously been discussed by Webster and Feller, [6] who highlighted that knowledge of patient expectations allows for the tailoring of appropriative advice to support injury management decisions and preparation for ACLR. It is commonly acknowledged that patient expectations of ACLR treatment are high and do not mirror reported outcomes. [4, 6, 55] However, results from this review suggest that those with low expectations and motivation to resume preinjury levels of activity are less likely to return. Clinicians should therefore aim to improve the expectations and motivation levels of patients with low scores but be cautious no to enhance these above that which is realistic.

Factors with variable results from this review that could also be considered during preoperative treatment include improving overall physical activity level through exercise prescription, lowering weight/BMI with dietary advice and nutritional support where appropriate and delivering a shorter time to surgery.

Another factor identified to be predictive of returning to physical activity was a patella tendon graft (BPTB) in favour of a hamstring graft. The graft type of choice is most commonly based on surgeon recommendation with mixed results reported in the literature for the optimum graft choice. [56] There are a number of options available with most recent recommendations suggesting the choice to be made individual to each patient to match their anatomy, age, needs and expectations. [57] This research adds to the discussion that a BPTB may be more likely to support a RTPA.

This review also highlighted the number of outcome measures used in ACL rehabilitation to assess RTPA. Five different outcomes were used across the eight studies to assess physical activity/sport engagement at four different postoperative time points. A recently published consensus statement highlighted this controversy in defining how and when we measure RTPA after ACLR and what is deemed to be a successful outcome. [58] Currently, no standard set of outcome measures exist (e.g. core outcome set) to allow for consistent reporting of ACLR outcomes in clinical practice. This is an important gap within the research.

### Clinical practice summary

The predictors identified in this review may support clinicians in focussing preoperative treatment to increase quadriceps strength through resistance training, assess and manage expectations and motivations to RTPA through education, discuss and consider a BTPB graft and have constructive conversations with patients about important lifestyle changes that may improve long-term outcomes.

### Future research summary

This review has highlighted a number of areas of focus for future research. Firstly, that further high-quality prospective predictor studies are needed that consider relevant confounding factors to determine preoperative predictor variables of ACLR outcomes. Secondly, determining an optimal preoperative treatment package and understanding the clinical implications of this. Finally, mirroring other recent research findings, a consistent set of outcome measures needs to be developed with an agreed time frame of assessment to determine the success of ACLR.

### Strengths and limitations

An inclusive approach was taken allowing for the inclusion of all study designs, outcome measures of interest and time-points of assessment. A risk of bias and certainty of evidence assessment was completed, and the initial search was updated closer to the time of submission to ensure newer studies were not missed. However, five of the eight studies included in this review were scored as ‘high’ on the risk of bias assessment with an overall very low-quality of evidence using the GRADE framework. The overall population group included in this review was dominated by males (> 70%), despite females being at a greater risk of sustaining an ACL injury. [59] However, despite a greater risk, incidence is reported in the literature to be higher in males which offers a potential explanation for their dominance across the study population. Further consideration is however, needed for female ACL injuries. A 2016, 21-year population-based study highlighted that incidence of injury has remained stable in females despite significantly decreasing over time in males. One explanation for this could be due to increased prevention strategies adopted by males, however, as we become increasingly more aware of the gender gap in medical research, it is important to consider that the male-heavy ACL evidence base may not directly apply to female patients. Considering sex and injury alone does not fully consider the biological, psychosocial and environmental differences which may add to the injury picture. [56]

In addition, we were unable to complete a meta-analysis as data were deemed to be heterogeneous, which limits robust analysis of the results. Further, four of the final eight studies were identified through reference list searching and so, there is the potential that further relevant studies may have been missed.

## Conclusion

Very-low level evidence supports the use of four variables (quadriceps strength, psychovitality, patients’ estimation of their ability to return to their preinjury level and graft choice) as predictors of return to physical activity after ACLR.

Further low-risk studies are required to support our understanding of the identified predictors in this study and to add breadth in identifying further variables. Further work is also needed to support the development and delivery of prehabilitation packages that address the identified risk factors to determine if this subsequently improves outcomes and to provide consistency in the reporting of ACLR outcomes.

## Electronic supplementary material

Below is the link to the electronic supplementary material.

Supplementary Material 1

Supplementary Material 2

## Data Availability

All data generated or analysed during this study are included in this published article [and its supplementary information files].

## Abbreviations

ACL: Anterior Cruciate Ligament
ACLR: Anterior Cruciate Ligament Reconstruction
AFLMOA: American Football League Medical Officers Association
BMI: Body Mass Index
BPTB: Bone-Patella Tendon-Bone
GRADE: Grading of Recommendations Assessment, Development and Evaluation
IKDC: International Knee Documentation Committee
K-SES: Knee Self-Efficacy Scale
MeSH: Medical Subject Heading
NHS: National Health Service
PAS: Physical Activity Scale
PEOS: Population-Exposure-Outcome-Study
QUIPS: Quality in Prognostic Studies
ROM: Range of Movement
RTPA: Return to Physical Activity
RTS: Return to Sport
