# Supplementary material for: Preoperative predictors for return to physical activity following anterior cruciate ligament reconstruction (ACLR): a systematic review
Source: BMC Musculoskelet Disord. 2023 Jun 9;24:471. doi: 10.1186/s12891-023-06489-5 (PMC10250179; doi:10.1186/s12891-023-06489-5)
Supplement: Supplementary file 2 — Supplementary Material 2 [file 12891_2023_6489_MOESM2_ESM.docx]

### Supplementary File 2, Overview of predictive factors

| Predictive | Not predictive | Variable |
| --- | --- | --- |

| **Category** | **Predictive Factor** | | **Predictor** |
| --- | --- | --- | --- |
| **Physical** | Concentric quadriceps torque | | Yes^21^ |
|  | Physical activity rating scale | Tegner | Variable  (yes^34, 25, 31, 39^, no^32, 39^) |
|  |  | Noyes | No^32^ |
|  |  | Marx | No^34^ |
|  | Knee specific patient reported outcome measure | Lysholm | No^32^ |
|  |  | IKDC |  |
|  |  | Knee function^a^ | No^33^ |
|  | Passive knee range of motion | | No^33^ |
|  | Eccentric quadriceps torque | |  |
|  | Concentric and eccentric hamstrings torque | |  |
|  | Anterior knee pain | |  |
| **Psychosocial** | Psychovitality questionnaire  (score ≥15) | | Yes^32^ |
|  | Estimation of ability to return to preinjury level | | Yes^38^ |
|  | K-SES_Present_ | | Variable  (yes^39^, no^39^) |
|  | K-SES_Future_ | | No^39^ |
|  | Patient estimation of time (number of months) to achieve postoperative markers | | No^38^ |
|  | Goal to return to preinjury level | |  |
| **Demographic / Clinical** | Graft type – Bone-Patella Tendon-Bone | | Yes^33^ |
|  | BMI | | Variable  (yes^35^, no^33^) |
|  | Weight | | Variable  (yes^36^, no^33^) |
|  | Time to surgery | | Variable  (yes^33^, no^34^) |
|  | Smoking status | | No^34^ |

International Knee Documentation Committee (IKDC); Knee-Self Efficacy Scale (K-SES); Body Mass Index (BMI)

^a^Two questions from IKDC
